# Role of Platelet Concentrates in Dental-Pulp Regeneration: A Systematic Review of Randomized Clinical Trials

**DOI:** 10.30476/dentjods.2023.96000.1912

**Published:** 2024-06-01

**Authors:** Zahra Kiaipour, Mahdieh Shafiee, Ghassem Ansari

**Affiliations:** 1 Postgraduate Student, Dept. of Pediatric Dentistry, Dental School of Shahid Beheshti University of Medical Sciences, Tehran Iran; 2 Stem Cell Research Canter, Tissue Engineering and Regenerative Medicine Institute, Tehran Central Branch, Islamic Azad University, Tehran, Iran; 3 Head of the Hospital Dentistry and Sedation Unit, Dept. of Pediatric Dentistry, Past-President of IADR-Iranian Division, Dental School Shahid Beheshti Medical University, Tehran, Iran

**Keywords:** Dental Pulp, Regeneration, Platelet Concentrates, Platelet rich plasma, Platelet rich fibrin, Injectable Platelet rich fibrin

## Abstract

**Statement of the Problem:**

Treatment of immature necrotic teeth is a problematic situation. Conventional root canal therapy is challenging and leaves a weak, fragile, and undeveloped tooth for lifetime.

**Purpose:**

This review was aimed to assess the outcome of available randomized clinical trials (RCTs) on the efficacy of platelet concentrates (PC) in dentine-pulp complex regeneration.

**Materials and Method:**

In this systematic review, an electronic search was conducted on MEDLINE, EMBASE, Cochrane, and Google scholar databases. A further manual search was performed on the list of related articles in order to ensure inclusion of potentially missed articles in earlier electronic search. Those proved RCTs matched with the standard criteria were included following an initial assessment of abstracts and the text independently by the reviewers.

**Results:**

From the total 602 harvested articles, only 13 met the criteria and were evaluated with 11 having parallel design and 2 split mouth. Only one study featured low risk of bias, while three had moderate risk and the rest were at high risk of bias. Six studies had used platelet rich plasma (PRP), 4 employed platelet rich fibrin (PRF), one utilized injectable platelet rich fibrin (I-PRF), and three used both PRF and PRP for their experimental groups while blood clot (BC) was used as the control group for all. The success rate was reported at 87.3% judged by the absence of pathologic signs and symptoms.

**Conclusion:**

Dentin wall thickening, root lengthening and apex closure were higher in PC groups, however, these differences were not statistically significant in reported studies. It can be concluded that PCs promote the pulp tissue revitalization and continuation of root development. However, a consensus on its potency for true pulp regeneration is yet to be reached.

## Introduction

Treatment of necrotic or irreversibly inflamed immature pulp is considered as one of the most challenging situations in endodontic therapies. Due to the nature of thin underdeveloped roots of such teeth, they become more fragile with poor in their treatment prognosis. Accordingly, open apices along with thin dentin walls make the root canal treatment more complicated [ 1 ]. In this regard, more recent studies had focused on tissue engineering, which is believed to provide revitalization of the pulp tissue along with the root development continuation process [ 2 - 5 ]. A true regenerated pulp is agreed as being achieved when the new pulpal tissue is harvested by all its major components including nerves, vessels and cells. Odontoblasts are amongst the most important components of dental pulp tissue involving in root development as the most critical part of dentin-pulp complex regeneration [ 3 ]. As widely acknowledged, three major components of tissue engineering include scaffolds, healing promoter factors, and stem cells [ 4 ]. For harvesting true tissue regeneration, stem cells have to migrate or being grafted into the defective area. Scaffolds provide a three dimensional environment for cellular activities under the control of healing promoter factors [ 4 ]. 

Healing promoter factors include a wide variety of bioactive molecules such as growth factors, glycosaminoglycans, large groups of drugs, small molecules, and some inflammatory factors that regulate cellular behaviour [ 5 ]. Several growth factors are involved in regeneration of the dentine-pulp complex including bone morphogenic proteins, transforming growth factors, insulin-like growth factors, and vascular endothelial growth factor [ 6 - 7 ]. Scaffolds provide a three dimensional microenvironment for cell attachment, migration, proliferation, and differentiation [ 8 ].

Platelet concentrates (PCs) are autologous scaffolds providing several curtail growth factors and cytokines [ 9 ], which are released gradually in target tissue, enhancing the regeneration of various tissues including dentine-pulp complex. They have a significant role in cellular differentiation [ 10 ]. On the other hand, the release of fibrin, fibronectin and vitronectin from platelets will support cell migration and attachment [ 6 - 7 ]. Availability of natural growth factors in PCs will eliminate the need for expensive synthetic growth factors. Several earlier studies stated that the PCs have some degrees of anti-inflammatory effects facilitating regeneration process [ 8 , 11 ]. 

Platelet concentrate is obtained from whole blood centrifuge, which creates a highly concentrated platelet layer. Different types of PCs are achieved through various production protocols with different structural and regenerative properties. PCs are mainly categorized in three generations including platelet rich plasma (PRP), platelet rich fibrin (PRF), and injectable platelet rich fibrin (I-PRF) [ 12 ]. There are also a few other variations of PCs, which include platelet pellet (PP) and plasma rich in growth factors (PRGF) [ 13 ]. 

PRP is known as the first generation of PCs made through either one or two-step centrifuge process of peripheral whole blood with anti-coagulant [ 14 ]. In PRP, platelets are inactive and have a round shape, which are only activated after injection into the injured site upon exposure to injured collagen fibres [ 15 ] or by addition of coagulant factors such as bovine thrombin and/or calcium chloride to the solution to form platelet gel and release growth factors [ 16 ].

Choukroun *et al* [ 17 - 18 ] used a second generation platelet concentrate, PRF in a gel-form while it was harvested by a single centrifuge cycle of anticoagulant-free blood which did not require activation. 

I-PRF is a liquid form of PRF that achieved by a lower centrifuge speed in non-glass tubes. It provides an easier clinical application than PRF [ 19 ].

Several *in vitro*, animal, and clinical studies have shown the efficacy of different scaffolds in dentin-pulp complex regeneration. However, there are still controversies on the concept of an optimal and standard scaffold for regenerative endodontics [ 21 - 24 ]. Many animal and *in vitro* studies showed the positive impact of PCs on cell proliferation and differentiation [ 25 - 27 ], however only a few support these biomaterials as mediator for true regeneration. Odontoblasts are known as the most suitable cells of pulp tissue for regeneration purpose; while these cells were absent in many of the reported investigations, it was obvious to see that most of the newly formed tissues contained fibrosis and cementum like tissues [ 24 - 25 , 27 ]. Although histologic assessments were not attainable in reviewed clinical studies, these studies could only suggest a standard guideline based on the outcome of clinical and radiographic evaluations for future clinical applications. In this respect, the main success criteria in most clinical studies include radiographic evidence of root lengthening, dentin wall thickening, apical closure, periapical lesion healing with no remaining or new pathologic clinical signs, and symptoms with clear response to vitality pulp tests.

This systematic review was aimed to assess the outcome of most recent randomised clinical trials (RCTs) in respect to the efficacy of PCs in regeneration of the dentine-pulp complex. 

## Materials and Method

### Protocol development

This systematic review was designed based on the PRISMA guideline and registered by PROSPERO #CRD4-2022329487.

### Information source and search strategy

An electronic search was conducted on the four main databases of MEDLINE, EMBASE, Cochrane, and Google scholar. The keywords and phrases that were used included "platelet", "platelet concentrate", "platelet rich plasma", "platelet rich fibrin", "plasma rich in grows factor", "injectable platelet rich fibrin", "PRF", "PRP", "I-PRF", "PRGF", "regeneration", "endodontic", "dentine pulp complex", " revitalization” in this electronic research alone or in combination by means of Boolean operators include "AND", "OR" and "NOT". Additionally, a further manual search was conducted on the list of related articles obtained in order to identify and include any potentially missed article in earlier electronic search. The search period was limited to 2000 to October 2022. 

### Eligibility criteria and study selection

Those repeatedly marked articles from different sources were excluded and only counted as one assessed. At the initial stage, two reviewers assessed the titles and abstracts of the articles found in search in an independent manner looking for the relevance of each manuscript. Full text of the selected articles were then obtained and evaluated based on the inclusion criteria to be included in the study. The inclusion criteria were defined as RCTs with a standard consort based protocol and methodology that compared PC with blood clot (BC) as the standard scaffold accepted in regenerative endodontics in immature teeth. These studies had used the PC only in full pulpectomized immature teeth and not in those partially pulpectomized. Case reports, and case series were excluded with only RCTs being confirmed as the approved articles and evidence. In addition, only English language papers were included, which were published from 2000 to October 2022. Any disagreement during this process was resolved in consultation with a third reviewer. 

### Data collection and data items

Data were extracted independently by two reviewers (ZK and MSh), which included the study design, sample size, age, tooth type, type of injury, type of PC, PC preparation methods, centrifuge steps and duration, type of anticoagulant and activator, pre-treatment signs and symptoms, maturation level of the teeth, intra-canal medication, root canal disinfection method, sealing agent, and follow-up time.

The outcomes were extracted and evaluated based on presence or absence of signs and symptoms, response to pulp sensitivity tests, radiographic signs of healing in periapical lesion, root lengthening, apex closure, and dentinal wall thickening.

### Quality assessment of the individual studies

Two independent reviewers assessed the risk of bias to approve the validity of selected studies. The assessment was based on Cochrane Collaboration’s risk of bias assessment which includes random sequence generation (selection bias), allocation sequence concealment (selection bias), blinding of outcome assessment (detection bias), incomplete outcome data (attrition bias), selective outcome reporting (reporting bias), clear definition of inclusion, exclusion and success criteria, and sample size calculation. Blinding the participants and personnel (performance bias) was not possible in selected studies due to obvious the differences in steps of interventions. A score defined as “adequate”, “unclear” or “inadequate” was given to each study. Subsequently, the studies were scored as “low risk” if all criteria were adequate, “moderate risk” if one or more criteria were unclear, and “high risk” if one or more criteria were inadequate. 

## Results

A total of 602 articles were selected in this study with their titles and abstracts being assessed at the initial step. Subsequently, 581 articles were excluded since 224 were duplicates, 201 not directly relevant to endodontic regeneration, 33 *in vitro* studies, 27 reviews, 51 case reports or case series and 45 were animal studies. Among the remaining 21 RTCs, eight were excluded after their full text being reviewed as they lack inclusion criteria (five did not have BC as control and three had non-comprehensive methodology report). Finally, 13 articles were included with full material being used for data collection [ 11 , 26 , 28 - 38 ]. 

From the total 13 clinical trials included in the present review, 11 were parallel design while two were split mouth. Findings indicated that one study was at a low risk of bias [ 36 ], while three were at a moderate risk [ 11 , 33 - 34 ], and the rest were at high risk of bias (Table 1). All included studies were reported to have performed on necrotic pulps and single root immature teeth Tables 2). Six studies had used PRP, four used PRF, and two used both PRF and PRP, one used I-PRF, and one study used PRP, PRF and PP for their experimental groups (Tables 2). A series of investigations reported to have used other adjutant agents in combination with PCs. 

**Table 1:** Risk of bias assessment

	Random sequence generation (selection bias)	Allocation concealment	Blinding outcome assessor	Selective reporting (reporting bias)	Clear definition of inclusion and exclusion criteria	Complete outcome data	Sample size calculation	Risk of bias
Jadhav *et al*. 2012 [ 28 ]	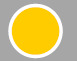	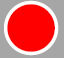	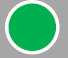	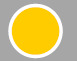	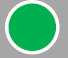	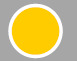	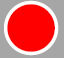	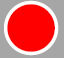
Bezgin *et al*. 2015 [ 29 ]	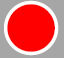	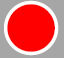	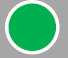	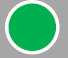	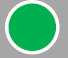	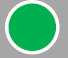	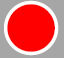	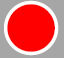
Narang *et al*. 2015 [ 30 ]	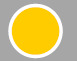	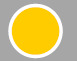	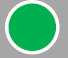	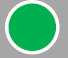	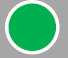	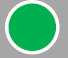	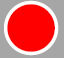	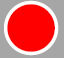
Lata *et al*. 2015 [ 26 ]	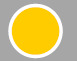	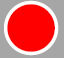	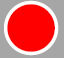	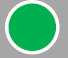	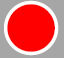	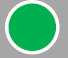	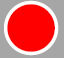	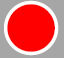
Sharma *et al*. 2016 [ 31 ]	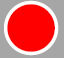	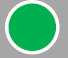	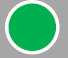	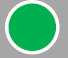	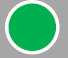	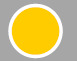	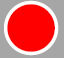	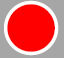
Algal *et al*. 2017 [ 32 ]	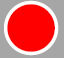	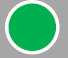	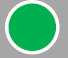	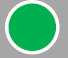	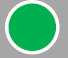	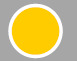	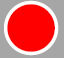	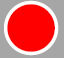
Shivashankar *et al*. 2017 [ 33 ]	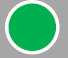	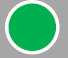	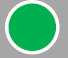	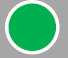	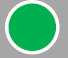	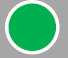	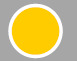	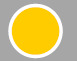
Ragab *et al*. 2019 [ 34 ]	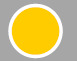	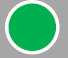	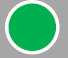	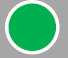	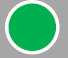	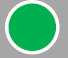	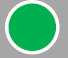	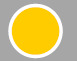
Rizk *et al*. 2019 [ 35 ]	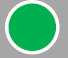	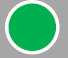	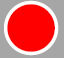	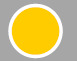	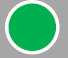	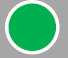	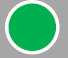	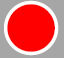
Ulusoy *et al*. 2019 [ 37 ]	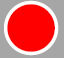	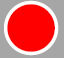	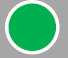	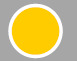	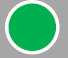	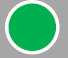	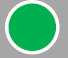	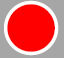
ElSheshtawy *et al*. 2020 [ 11 ]	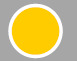	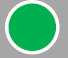	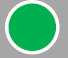	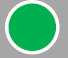	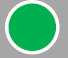	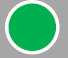	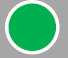	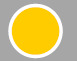
Rizk *et al*. 2020 [ 36 ]	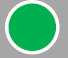	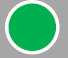	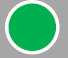	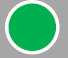	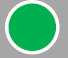	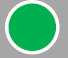	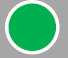	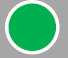
Nara *et al*. 2021 [ 38 ]	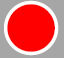	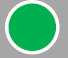	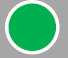	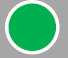	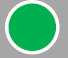	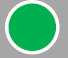	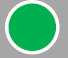	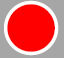

**Table 2:** Clinical trials: Study design, sample size, treatment groups and treatment strategy

Clinical trials	Sample size	Age	Teeth type	Injury type	Treatment groups	Irrigation	Instrumentation	Inter-canal medication	Sealing
Jadhav *et al*. 2012 [ 28 ]	20	15-18	Central incisors	Non-vital teeth	BC†	NaOCl 2.5%	Minimal	TAP***	IRM
					PRP‡+ collagen+ BC				
Bezgin *et al*. 2015 [ 29 ]	20	7-13	Single root teeth	Non-vital teeth with or without apical periodontitis		NaOCl 2.5%	No	Metronidazole/ciprofloxacin/ cefaclor	MTA¦
					BC	CHX†† 0.12%			
					PRP	Sterile saline			
						EDTA‡‡ 5%			
Lata *et al*. 2015 [ 26 ]	20	16-25	incisors	Non-vital teeth with or without apical periodontitis	BC	NaOCl 5.25%	NR	Ledermix	MTA
					PRP	Saline			GIC‡‡‡
									Composite
Narang *et al*. 2015 [ 30 ]	20	20>	NR	Non-vital teeth with or without apical periodontitis	BC	NaOCl 2.5%	Minimal	TAP	GIC
					PRP+ collagen				
					PRF§				
					MTA				
Sharma *et al*. 2016 [ 31 ]	16	10-25	Anterior maxillary teeth	Necrotic pulp with or without apical periodontitis	BC	NaOCl 2.5%	Minimal	TAP	GIC
					PRF				
					Collagen				
					PLGA**				
Alagl *et al*. 2017 [ 32 ]	30	9-11	Single root teeth	Non-vital teeth with or without apical periodontitis		NaOCl 2.5%	no	TAP	
					BC	Saline			MTA
					PRP	CHX 0.12%			GIC
						EDTA 17%			Composite
Shivasakhand *et al*. 2017 [ 33 ]	60	6-28	Anterior teeth	Non-vital teeth	BC	NaOCl 5.25%	Minimal	TAP	NR
					PRP				
					PRF				
Ragab *et al*. 2019 [ 34 ]	22	7-12	Maxillary anterior teeth	Traumatized necrotic teeth	BC	NaOCl 5%	NR	DAP †††	MTA
					PRF	Saline			GI
Rizk *et al*. 2019 [ 35 ]	30	8-14	First central incisors	Non-vital teeth	BC	NaOCl 2%	Minimal	TAP	MTA
					PRP	EDTA 17%			GI
									Composite
Ulusoy *et al*. 2019 [ 37 ]	88	8-11	Maxillary incisors	Traumatized necrotic teeth	BC	NaOCl 1.25%	No	Metronidazole/ciprofloxacin/ clindamycin	MTA
					PRP				GI
					PRF				Composite
					PP	Saline			
Elsheshta-wy *et al*. 2020 [ 11 ]	26	7 and younger	Anterior teeth	Traumatized necrotic teeth	BC	NaOCl 5.25%	Minimal	TAP	MTA
					PRP	Saline			Reinforced GI
						EDTA 17%			Composite
Rizk *et al*. 2020 [ 36 ]	30	8-14	Incisor teeth	Non vital teeth	BC	NaOCl 2%	NR	TAP	MTA
					PRF fragments	EDTA 17%			GI
									Composite
Nara *et al*. 2021 [ 38 ]	10	8-13	Single root teeth	Non vital teeth	BC	NaOCl 1%	Thoroughly	TAP	MTA
					I-PRF	EDTA 17%			GI
									Composite

* : Not reported,

† : Blood clot,

‡ : Platelet rich plasma,

§ : Platelet rich fibrin,

¦ : Mineral trioxide aggregate,

** : Poly lactic-co-glycolic Acid,

†† : Chlorhexidine,

‡‡ : Ethylenediaminetetraacetic acid,

*** : triple antibiotic past (metronidazole, ciprofloxacin and minocycline),

: ††† Intermediate restorative material, DAP: double antibiotics past,

‡‡‡ : Glass ionomer cement.

Ragab *et al*. and Rizk *et al*. [ 34 - 36 ], used bleeding induction in addition to PCs while Narang *et al*. [ 30 ] used collagen sponge in combination with PCs. Jadhav *et al*. [ 28 ] used metronidazole-containing collagen sponge as an inductive material, which enhanced revascularization, facilitated PRP placement into the canals and reduced infection prevalence as reported. This was the only study that reported platelet concentration values (106 platelets per each microliter of PRP).

One of the most important steps in regenerative endodontics, which could have affected on the outcome, was the canal disinfection methods. All studies used sodium hypochlorite as the major canal irrigators (Table 2). Ethylene-diamine-tetra-acetic acid (EDTA) 17% was the next prevalent irrigator in canal preparation procedure. Bezgin *et al*. [ 29 ] and Alagl *et al*. [ 32 ] used chlorhexidine as an additional antiseptic agent. 

Regenerative endodontic is a two-step treatment in which there is a need for inter-canal medication placement after the first session, in order to prevent bacterial regrowth and provide complete disinfection [ 15 ]. According to these reviewed studies, the most popular (61%) inter-canal medication was triple antibiotic past (TAP) which contains metronidazole, minocycline, and ciprofl-oxacin [ 11 , 28 , 30 - 33 , 35 - 36 , 38 ]. There are modifications in this mixture introduced by Ulusoy *et al*. [ 37 ] who used clindamycin while Bezgin *et al*. [ 29 ] used cefaclor instead of the minocycline part. Furthermore Ragab *et al*. [ 34 ] used double antibiotic past containing metronidazole and ciprofloxacin, without minocycline. Lata *et al*. [ 27 ] used Ledermix® Paste (RIEMSER, Greifswald, Germany) as the intracanal medicine paste, which has both anti-inflammatory and antimicrobial effects while it inhibits resorption in traumatized teeth. It is widely acknowledged that a perfect coronal seal is crucial for regeneration success by protecting the canal environment form bacterial re-infiltration and reinfection [ 32 ]; interestingly eight studies used double seal approach (Table 2). All included RCTs had a follow up skim of 12 months for their participants except Ulusoy *et al*. [ 37 ] with 27 months and Bezgin *et al*. [ 29 ], Nara *et al*. [ 38 ] and Narang *et al*. [ 30 ] with 18 months.

Since there is a need for anticoagulants for PRP preparation, calcium citrate and acid citrate dextrose were the two common anticoagulants used in the reviewed trials. In addition, PRP needs to be activated by activators such as calcium chloride and/or bovine thrombin. Bezgin *et al*. [ 29 ] used bovine thrombin for PRP activation; bovine thrombin is an immunogenic material, which can cause adverse reactions such as haemorrhage, thrombosis, and substantial immune reactions such as systemic lupus erythematosus [ 39 ]. In contrast, activators such as CaCl_2_ are believed to be able to resolve these problems [ 28 ].

### Clinical and radiographic outcomes of PRP

Jadhav *et al*. [ 28 ] reported clearly improved apical closure, periapical healing and dentinal wall thickening in PRP groups compare to that of BC groups; however, these results were not significantly different in terms of the root lengthening. Interestingly Lata *et al*. [ 26 ] reported significantly higher root lengthening in the PRP group when compared to the BC group. Vitality tests showed no significant differences between two groups, as was the case for periapical healing, apical closure, and dentin wall thickening [ 26 ]. El-Sheshtawy *et al*. [ 11 ] reported no significant difference in radiographic findings between PRP and BC groups as both groups failed to respond to vitality tests.

Alagl *et al*. [ 32 ] reported higher root lengthening in the PRP group compared to BC group. Bezgin *et al*. [ 29 ] stated that PRP achieved more favourable results in clinical and radiographic criteria than BC group. However, the differences were not statistically significant (*p*> 0.05). Rizk *et al*. [ 35 ] compared PRP with BC and illustrated promising results for PRP in radiographic root development criteria compare to that of the BC group. Vitality tests were reported as being negative in all specimens.

### Clinical and radiographic outcomes of PRF

Sharma *et al*. [ 31 ] compared PRF, BC, and collagen as scaffold for pulp regeneration and reported that PRF and collagen achieved better results in periapical healing, apical closure, and dentinal wall thickening; however, root lengthening was not favourable in the PRF group (Table 3). Ragab *et al*. [ 34 ] compared BC and PRF in apex closure, root lengthening and periapical healing but did not find any statistically significant difference between the two groups. Rizk *et al*. [ 36 ] compared PRF with BC and demonstrated better results for PRF in radiographic root development criteria when compared to BC group. Vitality tests were shown to be negative in all specimens. Nara *et al*. [ 38 ] utilized I-PRF in the experimental group and stated that I-PRF could enhance root lengthening more than BC markedly, although this was not about the case in periapical healing and dentin wall thickening.

**Table 3:** Main results of clinical studies

Clinical trials	Fallow up time (month)	Treatment group	Response to vitality test		Periapical healing			Apex closure			Root lengthening			Dentine wall thickening		
			Yes	No	Excellent	Good	Fair	Excellent	Good	Fair	Excellent	Good	Fair	Excellent	Good	Fair
Jadhav *et al*. 2012 [ 28 ]	6, 12	• BC*														
		• PRP†+ collagen+ BC	NR¦		-	70	30	10	60	30	-	60	40	-	30	70
					50	40	10	70	30	-	40	50	10	30	50	20
Bezgin *et al*. 2015 [ 29 ]	3, 6, 12, 18	• BC	20	80	30	10	60	60	-	40	NR			10	20	70
		• PRP	50	50	30	10	60	70	-	30				-	40	60
Lata *et al*. 2015 [ 26 ]	1,6,12	PRP	20	80	-	70	30	-	60	40	Cannot extract data			-	50	50
		• BC	20	80	-	80	20	-	40	60				-	20	80
Narang *et al*. 2015 [ 30 ]	6, 18	• BC	NR		-	60	40	-	65	35	-	60	40	-	50	50
		• PRP+ collagen			-	80	20	-	60	40	-	40	60	-	20	80
		• PRF‡			98	2	-	-	40	60	99	1	-	60	40	-
Sharma *et al*. 2016 [ 31 ]	6,12	• BC	NR		25	50	25	-	75	20	-	75	25	-	50	50
		• PRF			75	25	-	50	50	10	-	-	100	-	75	25
		• Collagen			25	75	-	50	25	7	-	25	75	25	50	25
		• PLGA§			-	25	75	-	50	50	-	50	50	-	25	75
Algal *et al*. 2017 [ 32 ]	3, 6, 9, 12	• BC	40	60	76	15	9	-	53	47	Cannot extract data			NR		
		• PRP	86	14	75	16	9		93	7						
Shivash-ankar *et al*. 2017 [ 33 ]	3,6,9,12	• BC	13.3	86.7	Cannot extract data			Cannot extract data			26.7	60	13.3	20	73.3	6.7
		• PRP	15.8	84.2							26.3	47.4	26.3	26.3	57.9	15.8
		• PRF	15	85							30	35	35	30	40	30
Ragab *et al*. 2019 [ 34 ]	6,12	• BC	NR		Not significant			Not significant			Not significant			NR		
		PRF														
Rizk *et al*. 2019 [ 35 ]	3,6,9,12	• BC		100	PRP significantly better			PRP significantly better			PRP significantly better			PRP significantly better		
		• PRP		100												
Ulusoy *et al*. 2019 [ 37 ]	27	• BC	100	0	100	0	0	Not significant			Not significant			Not significant		
		• PRP	100	0	100	0	0									
		• PP	100	0	100	0	0									
		• PRF	100	0	100	0	0									
Elshesh-tawy *et al*. 2020 [ 11 ]	3,6,9,12	• BC	0	100	Not significant			Not significant			Not significant			Not significant		
		• PRP	0	100												
Rizk *et al*. 2020 [ 36 ]	3,6,9,12	• BC	0	100	PRF significantly better			PRF significantly better			PRF significantly better			PRF significantly better		
		• PRF fragment	0	100												
Nara *et al*. 2021 [ 38 ]	18	• BC	NR		Not significant			NR			I-PRF significantly better			BC significantly better		
		• I-PRF														

* : Blood clot,

† : Platelet rich plasma,

‡ : Platelet rich fibrin,

§ : Poly Lactic-co-glycolic acid,

¦ : Not reported.

### Comparison between PRF and PRP with BC

Narang *et al*. [ 30 ] compared the efficacy of both PRF and PRP with BC with their report indicating statistically significant difference between BC and PRP groups, while some other factors such as periapical healing, root lengthening, and dentinal wall thickening were the three greater items in the PRF group compared to control. Shivashankar *et al*. [ 33 ] did not find any statistically significant difference between PRP, PRF, and BC groups in clinical and radiographic outcomes. Ulusoy *et al*. [ 37 ] detected no significant difference between pulp regeneration in four groups of BC, PRP, PRF and PP based on their radiographic evaluations reports. Interestingly all the teeth in this study reportedly had positive response to the vitality tests with such positive response being achieved earlier in the platelet rich groups. 

## Discussion

Since pulp tissue survival is highly curtail for its preservation of vitality it is important to have a viable and reliable source in order to successfully attempt for any pulp regeneration. To achieve the true and optimal pulp tissue regeneration, it is necessary to have multiple critical factors including proper stem cell source, signalling factors, and scaffolds [ 40 ]. There are two approaches to supply such needed cells and that includes grafting *in vitro* cultured cells and cell homing. Among these, cell homing procedure involves no laboratory cell preparation step, and it is widely considered as cost effective and more predictable alternative to other stem cell-based approaches. In this technique, signalling factors induce cells to migrate into the targeted area throughout the scaffold [ 17 ].

PCs are widely considered as a natural biocompatible scaffold, containing high concentrations of various growth factors, which encourages cell migration, proliferation and differentiation when introduced [ 13 ]. PRF and PRP are the two most common PC forms employed in many studies and are among the choice materials for ongoing research. These biomaterials have their main difference on production protocols and histologic characteristics [ 16 , 41 ]. PRP, as the first generation of PCs, is usually produced by two-step centrifuge of whole blood in anticoagulant containing tubes. RRF is the second generation of PCs that requires only one-step centrifuge and do not need anticoagulants for production [ 16 , 41 ]. 

On the other hand, PRP needs activators to become functional while such activators are believed to cause sudden fibrin polymerization which in turn leads to an unfavorable rigid structure formation [ 16 , 41 ]. This high concentrate fibrin network is a rather less suitable platform for cells and growth factor entrapment [ 41 ]. However, the presence of physiologic thrombin in PRF provides a gradual polymerization with resultant equilateral fibrin junction that has a more flexible and elastic network making it more favorable for cell migration and growth factor entrapment. It is evident through the recent research outcomes that structural differences can lead to a longer term growth factor release (up to 28 days) in PRF compared to that of PRP which are mostly releasing their growth factor within the first 60 minutes and immediate application of PRF is necessary [ 1 , 7 , 41 ]. It is of note that bovine thrombin is routinely use as an activator to PRP which has an adverse effect by irritating the immune cells. This in turn will results in the release and exposure of antibodies to factors V, XI and thrombin that are essential for an active coagulation process [ 7 , 22 ]. These comparative parameters may be able to determine the superiority of PRF as a regenerative agent. However, the liquid form of PRP makes it more user-friendly compared to the gel form PRF delivery system. In this line, the manufacturers have produced the I-PRF, which is a liquid modification of PRF and makes ease of their application. I-PRF has demonstrated the ability to release higher concentrations of various growth factors up to 10 days when compared to liquid PRP [ 12 ]. 

Although PRF gel application is quite challenging for this type of investigations, some studies made the use of PRF gel in the form of a membrane through squeezing while others have cut them into small fragments. It is important to note that the process of PRF squeezing is forbidden to be done on a piece of gauze as it absorbs in most of the growth factor rich PRF extract, but a two metal plate is best for this purpose [ 42 ].

Lolato *et al*. [ 48 ] stated that higher dentin wall thickening and root lengthening in platelet rich groups compared to BC groups are reported in earlier studies used in their systematic review; however, no statistically significant differences were shown in apex closure and periapical healing between groups [ 48 ]. On the same line, Metlerska *et al*. [ 27 ] had concluded from another systematic review that the PCs are effective and widely accepted biomaterial for regenerative endodontic procedure and further root development. 

 Despite all available data, there is still a wide range of controversies between the results of presented research outcomes. In one group there was no significant difference between PCs and conventional regenerative endodontics (BC usage), and in another group there have been an obvious superiority for PCs compared to BC. This could be explained partly by the variations in details of their methodology of trials. Other influencing factors include canal disinfection protocols, drugs used, apex width, adjutant regenerative factors, patient's age, and coronal sealing materials.

Due to the deep penetration of microorganisms into dentinal tubules of an immature tooth, chemical disinfection is advocated over the use of mechanical instrumentation [ 35 ]. Wide variations of irrigators are reported as being used in recent studies with most reported to have used NaOCl as the major irrigation agent. However, there is still no consensus over the ideal concentration of NaOCl in this procedure. Some studies suggested a 5% concentration while others recommended 1-3% as the non-toxic concentration on regional stem cells [ 34 - 35 ]. The concept of subsequent rinsing the canal with saline solution after the use of other irrigators could help reduce toxicity [ 1 ]. In fact, ethylenediaminetetraacetic acid (EDTA) had been used as a reliable irrigation agent alongside chlorhexidine and saline for many years [ 1 ]. EDTA with the concentration of 17% could be considered as an ideal solution for final irrigation. It has been shown to be able to reduce periapical stem cell death substantially and therefore, increasing the adherence of stem cells to dentin walls by 2.2 folds through higher dentin wettability. In addition, EDTA has the capacity to disengage growth factors entrapped in dentin through its demineralizing properties, which in turn will promote cell differentiation and tissue regeneration [ 2 ].

On the other hand, triple antibiotic paste (TAP) is considered as a complementary agent for canal disinfection. It contains minocycline as one of the components of TAP, which may adversely cause discoloration on treated tooth. There were trials in which minocycline had been replaced with clindamycin or cefaclor [ 29 , 37 , 43 ]. On the other side, Rizk *et al*. [ 35 ] reported that TAP containing minocycline results in more dentin wall thickening along regeneration process because it diffuses more readily in dentin.

Since regenerative therapy requires rich blood supplies and pulp tissue adjacent to the dentinal walls lacks adequate blood circulatory support, it is strongly recommended to have a minimum 0.8 mm apical width in order to have a successful regeneration attempt [ 16 ].

Coronal seal is another important step advocated in almost every procedural attempt in endodontic therapies. More recently, MTA had been looked at as the material of choice following its excellent clinical performances. Some of MTA’s superior properties include high seal ability even in the presence of moist, calcium release for cell attachment and proliferation, mineralization induction, alkaline rich environment for antimicrobial activity, differentiation and migration assisting in hard tissue producing cells, and regulation of cytokine production [ 34 , 44 - 45 ]. 

It is verified that histologic proof is the most reliable way to obtain a true regeneration occurrence amongst all available methods. However, due to the irreversible nature of damage encountered by histologic preparation, such proof could not be provided easily in clinical trials. A single available case report in the literature [ 46 ] indicated the use of histologic evaluation following extirpation of the pulp of treated tooth with PRP due to the presentation of constant pain and sensitivity to cold after 14 months and before attempts for root canal therapy. Histologic examination of the removed soft tissue of the pulp revealed fibrosis with mild inflammation and no odontoblast cells [ 46 ]. Interestingly, this case could evidently indicate no relation between symptoms and histologic status of the pulp tissue observed under the microscope [ 46 ]. In addition, Martin *et al*. [ 47 ] had reported another case of a tooth that had been extracted after regenerative therapy because of an oblique fracture diagnosed later in process; it immediately was subjected to histologic examination. The fibrotic, irregular mineralized tissue containing cementum-like structure with osteoid was among the findings in absence of any odontoblasts and Hertwig’s epithelial sheath [ 47 ]. Del Fabbro *et al*. [ 49 ] looked on animal studies, which had assessed PRP for dentin-pulp complex regeneration and reported that PRP cannot truly regenerate necrotic pulp, confirmed by subsequent histologic investigations. 

In an overall view, the success rate was reported to be 87.3% based on the absence of any pathologic signs and symptoms. Most studies revealed that the use of PCs have been associated with more favourable root development (apical closure, root lengthening, and dentin wall thickening); however the differences between the tested groups were not statistically significant [ 11 , 34 , 37 ]. Interestingly, several investigations had pointed out that they have considered vitality test response as an assessment tool following regeneration therapy [ 26 , 29 , 32 - 33 , 37 ]. Rizk *et al*. [ 35 ] reported no response to vitality testing in all control and experimental cases which may be explained by the MTA coating layer acting as an insulator or even short follow up duration (insufficient for nerve generation) or root wall thickening by cementum like tissue deposition that lacks tubular structure. 

Another important point to note in this process is that the PCs are normally obtained from peripheral blood, which lacks adequate dentine-pulp specific growth factor. Therefore, true pulp regeneration is hard to be obtained when using PCs as it requires the addition of specific growth factors, pulp specific stem cells, and dentine formation guiding factors like hydroxyapatite as supplementary materials. 

Due to the heterogeneous comparisons and reported outcomes, meta-analyses were not conducted. The clinical/ methodological heterogeneity among included studies did not allow for the conduction of meta-analyses, which can be considered as a limitation of this study.

## Conclusion

This systematic review revealed that PCs are among the most preferred methods in pulp tissue repair processes known so far, which promotes the pulp tissue revitalization and continuation of root development, dentin wall thickening, root lengthening, and apex closure. A consensus on its potency for true pulp regeneration needs to be reached by more qualified trials. 

Conflict of Interest: The authors declare that they have no conflict of interest.
